# The Pocket Formulary

**Published:** 1852-04

**Authors:** 


*The Pocket Formulary.* By Henry Beasley. First American from the last London edition; corrected, revised and enlarged. Philadelphia: Lindsay & Blakiston, 1852, pp. 443.

A good book for reference, but too big for the pocket. In truth, however, no body carries a formulary in his pocket. Some American might probably carry one in his *hat*, if made of a proper shape. It is strange, that publishers have not provided books for this national receptacle of portables. The book will be useful on the table, however, as other dictionaries are. It will also be acceptable to apothecaries.

